# Acknowledgement to Reviewers of *Micromachines* in 2017

**DOI:** 10.3390/mi9010033

**Published:** 2018-01-17

**Authors:** Micromachines Editorial Office

**Affiliations:** MDPI AG, St. Alban-Anlage 66, 4052 Basel, Switzerland

Peer review is an essential part in the publication process, ensuring that *Micromachines* maintains high quality standards for its published papers. In 2017, a total of 362 papers were published in the journal. Thanks to the cooperation of our reviewers, the median time to first decision was 19 days and the median time to publication was 49 days. The editors would like to express their sincere gratitude to the following reviewers for their time and dedication in 2017:
Aas, MehdiLi, ZhanhuaAbate, AdamLiang, JinxingAbdel-Motaleb, Ibrahim M.Liang, XinAbdel-Rahman, EihabLiao, Jiunn-DerAbdul Samad, YarjanLim, JiseokAbelmann, LeonLima, RuiAbolhasani, MiladLin, Chi-YingAbou-Hassan, AliLin, GungunAbu Khater, MohammadLin, Jr-LungAgarwal, MangilalLin, Shu-pingAguilella-Arzo, MarcelLin, XueAhamed, Mohammed JalalLin, YangAhn, Chae HyuckLinzon, YoavAhn, SeungyoungLiu, Cheng-HsienAi, YeLiu, DonAkhter, MahbubLiu, LianqingAkram, Muhammad NadeemLiu, ShengAl Qubeissi, MansourLiu, TingtingAl-Bataineh, SameerLiu, TingyiAlbert, JacquesLiu, YuHaoAlexeev, IlyaLiu, Yung-tienAl-Jumeily, DhiyaLo, Kai-YinAlmoazen, HassenLo, Yu-hwaAlsaleem, FadiLópez De Lacalle, Luis NorbertoAmack, JeffreyLu, JianAmann, AndreasLu, MiaoAmiralian, NasimLucchetta, GiovanniAmit, IddoLung-Ming, LuAmstad, EstherLuo, JerryAn, JiaLuo, QuantianAnand, RobbynLuo, RongcongAref, Amir RezaLuo, Win-jetArrue, JonLuo, XiaolongArutinov, GariLyding, Joseph W.Assaf, TareqMa, Hsiao-KanAtaman, ÇağlarMacDonald, Brendan D.Athanasiou, ChristosMacdonald, NiallAttanasio, A.Mace, CharlesAu, Sam H.Macheret, SergeyAusländer, SimonMackay, RuthAvci, EbubekirMadani, AbbasAzar, Amin S.Madhukar, Yuvraj K.Aziz, MuhammadMadrenas, JordiBabu, S.V.Maeda, YusakuBagolini, AlviseMaffettone, Pier LucaBahk, Je-HyeongMager, DarioBaldacchini, TommasoMalinauskas, MangirdasBamiedakis, NikosManjare, ManojBaranski, MaciejManolakos, DimitriosBardaweel, HamzehMao, WenbinBasabe-Desmonts, LourdesMarcos, MarcosBasden, AlastairMargueron, SamuelBasu, AnirbanMarinello, FrancescoBasuray, SagnikMarinov, ValBauer, PeterMarkopoulos, AngelosBauer, RalfMarsh, Eric R.Belfiore, Nicola PioMartel-Foley, JosephBellantone, VincenzoMartinez, AndresBenardos, PanoriosMartinez, Nathaniel W.Benedetti, IvanoMartín-Fabiani, IgnacioBéra, Jean ChristopheMaruda, RadosławBeriáin, AndoniMassoudi, MehrdadBerthier, JeanMastrangeli, MassimoBertsch, ArnaudMathews, ScottBhaduri, DebajyotiMATSUO, ShigekiBianchi, ValentinaMatteucci, MarcoBieniek, TomaszMaurotto, AgostinoBiffi, Carlo AlbertoMcCain, Megan L.Bishay, Peter L.Mcginty, SeanBittner, AchimMeagher, RobertBlanche, Pierre-alexandreMedina-Sánchez, MarianaBlanes, LucasMeijer, Han E.H.Borboni, AlbertoMena, Oscar VazquezBormashenko, EdwardMeng, FanbenBourouina, TarikMeredith, NathanBowen, JamesMerlo, SabinaBrankovic, Stanko R.Merrin, JackBrousseau, EmmanuelMiah, Abdul HalimBrouzes, EricMichael, AronBruggi, MatteoMichelmore, AndrewBrunet, PhilippeMickelson, Alan R.Bucci, DavideMileti, GaetanoBunch, Joseph ScottMillet, Larry J.Burgard, MatthiasMirbozorgi, AbdollahCakmak, OnurMishina, YujiCaliano, GiosueMistura, GiampaoloCanetta, ElisabettaModica, FrancescoCao, ChangyongMohd-Yasin, FaisalCao, XudongMoraes, ChristopherCappelleri, DavidMorley, Nicola ACarrascosa, MercedesMoron, CarlosCartagena-Rivera, AlexanderMoses, Matthew S.Casadevall i Solvas, XavierMyers, OliverCasalino, GiuseppeNabavi, Seyed AliCasas Bedoya, AlvaroNagai, MoetCeylan, HakanNagao, MasayoshiChakraborty, SumanNakao, MasayukiChan, Leanne Lai-HangNama, NiteshChang, Chih-WeiNarayan, RogerChang, HoNasiri, NoushinChang, JiyoungNeethirajan, SureshChang, Pei-ZenNelatury, SudarshanChang, Robert C.Nelson, JohnChembo, Yanne K.Ng, AlphonsusChen, ChengpengNg, Chiu-OnChen, Chia-HungNg, Sum HuanChen, Chia-YuanNielsen, Michael PChen, ChihchenNikolic, KonstantinChen, JinjuNikumb, SuwasChen, Jyh-JianNisato, GiovanniChen, Kuan-NengNiu, RanChen, PengyuNosko, OleksiiChen, XiangzhongNovak, RichardChen, YuluO’Mahony, ConorCheng, Chao-MinObikawa, ToshiyukiCheng, Chiang-hoOchoa, ManuelCheng, Chun-HuOhlckers, PerCheng, Hung-LiangOk, Jong G.Cheng, YaOmar, ElmazriaChien, Ying-RenOmrani, EmadChinappi, MauroOnck, Patrick R.Ching, Tak-ShingOrtega-Casanova, JoaquínChiolerio, AlessandroOta, HirokiCho, Dong-WooOu, HaiyanChoi, Dong-HoonOu, JianzhenChoi, JungwookOuyang, MengxingChoi, SeokheunPacchierotti, ClaudioChoi, Seung-BokPaek, JungwookChoi, Yong GyuPagliara, StefanoChristoffer, AbrahamssonPakdelian, SiavashChu, JiaruPallaoro, AlessiaChuang, Cheng-hsinPan, HengChun, HongguPandey, SantoshCinti, StefanoPang, Da-ChenCitterio, DanielPani, DaniloClévy, CédricPantano, MariaCollard, DominiquePany, ThomasColtro, Wendell Karlos TomazelliPaoloni, ClaudioComi, ClaudiaParenti, PaoloConnelly, DanielPark, Byung-WookCooke, MichaelPark, HangueCordiner, StefanoPark, Jae SungCorigliano, AlbertoPark, Jang-UngCoskun, Ahmet F.Park, Je-KyunCostantino, LucaPark, Nam-GyuCoutu, RonaldPark, Yong-HwaCrane, NathanPark, Yun DanielCranford, StevenPassilly, NicolasCraw, PascalPatel, SaurinCretu, EdmondPatten, JohnCroissant, JonasPawashe, ChytraCybart, Shane A.Pei, AllenDagdeviren, CananPei, LingDai, JingPeiner, ErwinDaido, HiroyukiPerhinschi, MarioDarinskii, AlexanderPersano, LuanaDas, JagotamoyPersson, AndersDash, BirajaPhan, Hoang-PhuongDavies, Matthew A.Phang, Swee KingDe Dood, Michiel J.Piao, XianjiDe Fazio, MarcoPiekiel, Nicholas W.De La Fuente, Germán FranciscoPina, Maria PilarDe Sio, LucianoPiotto, MassimoDebliquy, MarcPIRO, BenoitDeiss, FrederiquePisco, MarcoDell'Olio, FrancescoPoenar, Daniel P.Delnavaz, AidinPokhrel, MadhabDeMiguel-Ramos, MarioPolavarapu, LakshminarayanaDemir, Ali GökhanPoletkin, KirillDeng, Nan-NanPonchak, George E.Desai, RaviPoot, MennoDeSimone, AntonioPopov, Krastimir BorisovDevendran, CitsabehsanPotma, EricDewulf, WimPradhan, Dhiren K.Di Falco, AndreaPriest, CraigDianat, PouyaPsimoulis, PanagiotisDias Soeiro Cordeiro, Maria NatáliaQamar, AfzaalDidar, TohidQi, HaitaoDijksman, J. FritsQian, KemaoDikin, Dmitriy A.Qian, ShizhiDiller, EricQian, YouDing, GuifuQian, ZhenyunDing, Xiaoyun (Sean)Qiao, RuiDinh, ToanQiu, ChengweiDobre, GeorgeQiu, YongqiangDoeven, EganQiu, ZhenDong, BinQu, HongweiDong, HanshanQuack, NielsDong, LiangQue, LongDong, LiliRabenorosoa, KantyDonley, ElizabethRahmani, RaminDosen, StrahinjaRais, RanaDrazer, GermanRajaraman, SwaminathanDrese, Klaus StefanRamini, AbdallahDu, E(Sarah)Ramos, DanielDu, LiqunRao, Prahalad KDuan, BinRao, SmithaDuffy, MaeveRaptis, IoannisDuncanson, Wynter J.Rasheduzzaman, MdDunsby, ChristopherRasponi, MarcoDusny, ChristianRaspopoulos, MariosDwyer-Joyce, RobRausch, PeterEhrmann, AndreaRebollar, EstherEinstein, Daniel R.Reboud, JulienElani, YuvalRees, AndrewElbuken, CaglarReigh, Shang YikElsayed, MohannadRejinold, SanojEmadi, ArezooRen, KangningEmberly, EldonRen, YongEnder, FerencRen, YukunEnikov, Eniko T.Rengel, RaulErdmann, AndreasRezk, AmgadEscobedo, CarlosRizzo, RoccoEsfandyarpour, RahimRobson, NinaEtienne, DagueRodrigo, Peter JohnEugenio, FazioRoper, MichaelFaivre, DamienRosengarten, GaryFalco, GianlucaRoth, JohannesFarah, ShadyRughoobur, GirishFarokhi, HamedRühmer, DennisFassi, IreneRyzhakov, PavelFaulkner-Jones, AlanSachid, AngadaFeiertag, GregorSaha, SourabhFeldman, MartinSaito, AkiraFemenía, Francisco CamarenaSalaoru, IuliaFernandes, ElisabeteSalter, PatrickFernández Romero, Juan ManuelSameoto, DanFerrante, CamillaSan Sangorrin, VictorFerraro, DavideSánchez De Rojas, JoseluisFerraro, PietroSánchez, Beatriz JuradoFerreira, Isabel M.M.Sandner, ThiloFirebaugh, SamaraSantiago, Ferrándiz BouFleischer, JürgenSantiago-Aviles, Jorge J.Flores-Arias, M.T.Santos, AbelFlorian Baron, CamiloSapienza, LucaFlowers, GeorgeSariyildiz, EmreFodor, PetruSarles, AndyFouillet, YvesScaini, DenisFoulds, IanSchaap, AllisonFournel, FrankSchenk, HaraldFrançais, OlivierSchertzer, MichaelFranzke, JoachimSchift, HelmutFriedt, Jean-MichelSchimpf, MartinFrisch, BenjaminSchmidt, MichaelFu, DanSchmidt, Oliver G.Fukuda, TakeshiScholten, KeeFurutani, KatsushiSchulz, RuthGabriel, Ellen Flávia MoreiraSchwaller, PatrickGad, AnnicaScotti, GianmarioGale, BruceSealy, MichaelGallacher, BarrySedlmeir, FlorianGao, WeiSenderowski, CezaryGarcía, Juan C.Senesky, DebbieGarcía-Cámara, BraulioSeok, HyunGau, Jenn-TerngSeok, Tae JoonGerber, DoronSepúlveda, NelsonGhassemali, EhsanSerry, MohamedGhoneim, Mohamed TSethu, PalaniappanGhosh, AyanjeetSeybold, JonathanGiacomello, AlbertoSeymour, JohnGiacomozzi, FlavioShah, LawrenceGiardini, ClaudioShao, LeiGielen, FabriceSharma, MrigankGilbert, DanielSharma, PankajGiraud, FredericSheen, Horn-jiunnGiunta, GaetanoShevchuk, I.V.Go, Jeung SangShevkoplyas, SergeyGoda, KeisukeShih, SteveGoel, SauravShikida, MitsuhiroGomez, FrankShikida, MituhiroGonzalez, FernandoShimizu, JunGordiichuk, PavloShintake, JunGotoh, YasuhitoShkunov, MaximGrecov, DanaSieber, IngoGreener, JesseSievenpiper, DanGrigsby, Christopher L.Siller, Hector R.Grosjean, ThierrySilva Girão, PedroGross, Jason N.Silvan, UnaiGrulkowski, IreneuszSim, Sang JunGspann, ThuridSing, Swee LeongGuan, WeihuaSingh, Surya P.Gui, LinSkylar-Scott, MarkGuijt, RosanneSo, HongyunGuijt, Rosanne M.Sohgawa, MasayukiGuna, JožeSøndergaard, ThomasGutruf, PhilippSones, CollinGuvendiren, MuratSong, DongHaitjema, H.Song, XuHan, ArumSridharan, M.Hanada, YasutakaStanley, ClaireHane, KazuhiroSteager, EdwardHans-Joachim, KrauseSteenson, David PaulHany, HassaninSterken, TomHao, GuangboSteward, Robert L.Haque, RezwanulStiharu, IonHaraldsson, TommyStratakis, EmmanuelHarnois, MaximeStreubel, RobertHarris, PeterSu, Quang T.Hassan, Sammer-ulSubiantoro, AlisonHatta, AkimitsuSugimura, JoichiHe, JiankangSul, OnejaeHe, MeiSun, ChenHe, XiaomingSun, MengHeise, RainerSun, YongHilber, WolfgangSung Kim, DongHiller, KarlaSun-Kyu, LeeHirai, YoshikazuSuzuki, KenichiroHirtz, MichaelSznitman, JosueHo, Megan Yi-PingTabib-Azar, MassoodHo, Mei-LinTaguchi, YoshihiroHo, Tsung-YiTaheri, ParsaHoettges, KaiTakemura, KenjiroHoffman, JasonTaklo, Maaike M. V.Hölscher, HendrikTan, SayHomsy, AlexandraTanabe, YujiHong, SukjoonTanaka, YoHopwood, Jeffrey A.Tang, William C.Hosseinian, EhsanTang, YuanHou, XiaodongTarantino, LauraHowlader, MatiarTarn, Mark DHoxley, DavidTassieri, ManlioHsu, Cheng-cheTatar, ErdincHsu, Wei-LunTay, Chor YongHsue, Albert Wen-JengTemiz, YukselHu, AnmingTenje, MariaHu, GuoqingThalhammer, GregorHu, JieThean, AaronHu, ZhongxuTichem, MarcelHua, MengTixier-Mita, AgnesHuang, HuTjahjowidodo, TegoehHuang, Kuo-ChengToal, VincentHuang, LiangliangToan, Nguyen VanHuang, Po-HsunToda, MasayaHuang, QijinToley, Bhushan J.Huang, Shyh-ChourTong, MingmingHuang, SunanTootkaboni, MazdakHuang, XianTorres, Pedro M. B.Huang, ZhaorongTortschanoff, AndreasHughes, Michael PycraftTowfighian, ShahrzadHung, Jung-ChouToy, RandallHung, WayneTrigona, CarloHunstig, MatthiasTsai, Chia-Hung DylanHuo, DehongTsamis, ChristosHutter, TanyaTsao, Chia-WenHwang, Chi-HungTseng, Victor Farm-GuooHwang, Suk-WonTseng, Wei-LungIannazzo, DanielaTseng, Wei-TsuIanoul, AnatoliTserepi, AngelikiIborra, EnriqueTsujii, ToshiakiIchiki, RyutaTsujino, SoichiroIde, KeisukeTsutsui, HideakiImani, Mohammadreza F.Tuohiniemi, MikkoImboden, MatthiasUchikoba, FumioIn, JungbinUhlmann, EckartIno, KosukeUnzueta, LuisIonescu, Elena RodicaUrban, GeraldIsaac, Kakkattukuzhy M.Uzunlar, ErdalIsozaki, AkihiroVallee, ChristopheJackson, NathanVan Dam, R. MichaelJain, AkhileshVan Erps, JurgenJalaal, MaziyarVan Vliet, LiisaJalabert, LaurentVanapalli, SrinivasJalili, RouhollahVázquez De Aldana, Javier RodríguezJambovane, SachinVazquez, Rebeca MartinezJanaideh, Mohammad AlVillani, MarcoJang, Jae-HyungVisconte, CarmenJavanmard, MehdiVlassov, SergeiJayamohan, HarikrishnanVoiculescu, IoanaJensen, Brian D.Volland, BurkhardJeong, Jae-WoongWada, YujiJeong, Ki-HunWaddell, EmanuelJeong, Sung HoWadsak, WolfgangJia, YuWaghmare, PrashantJiang, HaoWan, LeoJiang, JunhuaWan, MinJin, QinghuiWang, ChunjuJin, ZhongminWang, Dung-AnJing, WumingWang, HaoJohnston, IanWang, JunboJones, J CliffWang, LiliangJoung, Hyou-ArmWang, LiyuJuang, Jia-YangWang, PengJung, Hyun SukWang, PengfeiJung, HyungilWang, PengtaoKaajakari, VilleWang, ShueKACEM, NAJIBWang, ShuodaoKajimoto, HiroyukiWang, SihongKalantar-Zadeh, KouroshWang, Thomas D.Kale, AkshayWang, YouminKaler, KaranWang, ZhaomengKaltsas, GrigorisWang, ZhenfengKameoka, JunWark, Alastair W.Kanda, KensukeWarkiani, Majid EbrahimiKaneta, TakashiWasisto, Hutomo SuryoKang, Dong-KuWebster, J. G.Kang, DongyangWei, QingshanKang, Soon JuWei, TaoKang, YuejunWei, XueyongKar, ShantimoyWeigl, Bernhard HKaradimitriou, NikolaosWeinberg, MarcKaranassios, VassiliWelch, TreKärnfelt, CamillaWesterhausen, ChristophKassavetis, SpyrosWheeler, AaronKastner, JohannWhyte, GraemeKawahara, TomohiroWilliams, MartinKeating, AdrianWillmott, GeoffKeleher, Jason J.Winkler, AndreasKhabashesku, ValeryWiśniowski, PiotrKhalafallah, Alhossain A.Wolf, MartinKhan, MuhammadWong, Teck NengKhoshmanesh, KhashayarWoolley, Adam T.Kikuchi, HiroakiWu, Shin-TsonKillgore, JasonXianyu, YunleiKim, AlbertXie, YanboKim, Heung-KyuXiong, XingguoKim, Hong NamXu, FengKim, JaehwanXu, JianKim, KeekyoungXu, JiawenKim, KyunghoonXu, KunningKim, YongTaeXu, WeixingKirkko-Jaakkola, MarttiXu, YongKlotzkin, DavidYadavali, SagarKo, HyounghoYaish, Yuval E.Ko, SeungDeokYamanishi, YoKoKoh, Kah HowYan, JingKokkoris, GeorgeYan, StephenKoltay, PeterYang, ChaoyongKonozsy, LaszloYang, FengchangKootala, SujitYang, Ruey-JenKottapalli, Ajay Giri PrakashYang, RuiguoKoumoulos, EliasYang, ShengyuanKowalewski, TimothyYang, ShikuanKrick, Brandon AYang, ShoufengKrolczyk, GrzegorzYang, TieKubby, JoelYang, ZhengbaoKudryashov, Sergey I.Yao, Da-JengKumar, AnilYao, JunKumar, SandeepYapici, Murat KayaKumeria, TusharYeh, Li-HsienKurkuri, MahaveerYeo, JunyeobKwok, Chee YeeYeong, WaiLächelt, UlrichYoon, Eui-SungLaHaye, MatthewYoon, Hyeun JoongLake, Robert AYoon, Hyun C.Lam, David C.CYoshimura, TakeshiLam, Raymond H. W.Younis, MohammadLamberti, LucianoYu, ChunyangLancaster, RobertYu, Feiqiao BrianLancry, MatthieuYu, JiangboLang, UdoYu, KaiyanLaopoulos, TheodoreYu, YuanyuanLapizco-Encinas, BlancaYu, ZetaLarsen, TomYuan, JunqiLawrie, BenjaminYue, TaoLe Gac, SeverineZagnoni, MichelleLe Pioufle, BrunoZahn, Jeffrey D.Le Traon, OlivierZangheri, MartinaLeach, RichardZębala, WojciechLeccese, FabioZegarra Flores, JesusLee, Cheng-chungZenati, Marco A.Lee, Chia-YenZeng, HaoLee, Chung-HaoZergioti, IoannaLee, Eon SooZhai, LeiLee, Hyunjoo J.Zhang, EnxiaLee, HyunseopZhang, GuigangLee, JungchulZhang, LucyLee, JungwooZhang, NanLee, Sang-JoonZhang, RuiLee, SangminZhang, WuLee, Seok JaeZhang, XiaoyangLee, SeunghyunZhang, XiaoyuLee, Sung SikZhang, XumingLee, SunwooZhang, YiLee, Won ChulZhao, ChunLeem, Jung WooZhao, CunluLei, ShutingZhao, HongLelea, DorinZhao, HuiLesch, AndreasZhao, XinLevin, MichaelZhao, YulongLewpiriyawong, NuttawutZheng, BoLi, BeibeiZheng, JinchuanLi, Cheuk-WingZheng, YiLi, DeyuZhong, JingLi, HaijunZhou, JianLi, JianzhaoZhou, KunLi, JinxingZhou, LiLi, LongqiuZhu, YipingLi, Paul C.H.Zolin, LorenzoLi, XiaodongZuo, WenjieLi, Yangmin


